# Education Research: Enhancing Medical Student Interest in Careers in the Clinical Neurosciences Through a Hands-on Procedure Workshop

**DOI:** 10.1212/NE9.0000000000200010

**Published:** 2022-10-14

**Authors:** Collin Sanderson, Khadijah Mazhar, Mark P. Goldberg, Shilpa Chitnis, Ryan Hays, Hina Dave

**Affiliations:** From the Department of Neurology (C.S., K.M., S.C., R.H., H.D.), University of Texas Southwestern Medical Center, Dallas; and Department of Neurology (M.P.G.), University of Texas Health Science Center, San Antonio.

## Abstract

**Background and Objectives:**

It is predicted that the current shortfall of neurologists will continue to grow beyond current training rates. It is well documented that medical students often possess stigmatizing beliefs toward neuroscience-based careers. Preclerkship medical education is where many medical students lay the foundation for specialty interests, and at some medical schools, it may be their only direct exposure to neurology. Providing preclerkship students with exp osure to the unique aspects of clinical neuroscience such as procedures is a possible avenue for increasing student interest.

**Methods and Curriculum Description:**

We sought to assess the influence of a procedure workshop on student specialty interest. We organized a hands-on procedure workshop for preclerkship medical students to learn examination skills and procedures used by adult/pediatric neurologists, neurosurgeons, and psychiatrists. Twelve different stations were run by faculty, trainees, and technicians. Attendance was optional, and students were free to move between stations according to their time and interests. Most stations involved some brief education and time for students to practice or take part in the procedure. Attendees completed an exit survey on their retrospective interest in the relevant specialties before attending the workshop, prospective interest after attending the workshop, and the helpfulness of each station in understanding the procedure. Statistical analyses were performed on the survey responses to determine change in specialty interest resulting from the workshop.

**Results and Assessment Data:**

A total of 111 students attended the workshop, and 104 (94%) filled out the postsurvey. Most were from the second-year medical student class. Approximately 41% of the second-year class attended. There was an increase in student interest (*d* = 0.6346) in the clinical neurosciences by the Fisher exact test (*p* < 0.0001). Thirty-three attendees (32%) reported an increased interest in the specialties. Of the students who reported having no prior interest in the clinical neuroscience specialties, 82% (18/22) had an increased interest as a result of the workshop.

**Discussion and Lessons Learned:**

A hands-on procedure workshop improved medical student interest in the clinical neurosciences. Although its effect on future specialty choice is unclear, preclerkship experiences such as a procedure workshop may be a useful addition to medical school curricula to foster interest in neurology and the clinical neurosciences.

Neurology and psychiatry in the United States are experiencing a shortage of physicians, leading to long wait times and poor access to care. It is predicted that this shortage will continue to grow as the burden of neurologic disease rises with the aging population,^1^ and the number of providers being trained in these specialties continues to stagnate.^2,3^ Promoting medical student interest in these specialties is an important way to combat this growing shortage.

It is well documented that many medical students possess stigmatizing beliefs toward neuroscience-based careers. The 2 most well documented are as follows: (1) the belief that neuroscience is intimidating or complex, previously coined neurophobia,^4-6^ and (2) the belief that specialties like neurology diagnose disease but cannot treat it (diagnose and adios).^7^ These beliefs are often cited by physicians who chose specialties other than the neurosciences as reasons for their decision. Preclerkship education is where many medical students begin laying the foundation for interest in specialty choices, and at some medical schools, it may be their only direct exposure to the neuroscience. Physicians in the neurosciences often cite the quality of their preclerkship neuroscience curriculum and their interactions with neuroscience faculty as highly influential in their choice of specialty.^4,8-12^

Procedure-based specialties such as surgery have demonstrated the utility of hands-on workshops in increasing medical student interest in the specialty and confidence in performing tasks previously thought to be intimidating (e.g., suturing and laparoscopy).^13,14^ Medical students also demonstrated increased confidence in lumbar puncture after a brief hands-on training session.^15^ It has been suggested that similar hands-on opportunities in the neurosciences may promote interest in the neuroscience specialties,^9^ perhaps by reducing stigma, increasing student confidence in related procedures, and providing opportunities to interact with diverse faculty. However, there is currently little evidence to show whether hands-on neuroscience workshops actually affect medical student specialty interest and eventual career choice. The purpose of this study is to assess the influence of a hands-on neuroscience workshop on student interest in these specialties.

## Methods and Curriculum Description

The University of Texas Southwestern Student Interest Group in Neurology (SIGN), in collaboration with the student interest groups for Neurosurgery and Psychiatry, organized a hands-on multimodal procedure workshop for preclerkship medical students to learn examination skills and procedures used by adult and pediatric neurologists, neurosurgeons, and psychiatrists. The planning began in midsummer, and the event was scheduled in October during the preclerkship neurosciences course with oversight from the neurosciences course directors and the office of medical education. Attendance was optional. The workshop was situated in the medical student's team-based learning center, next to another required class activity for convenience and to encourage attendance. Invitations to the event were sent to the medical student class by email, and verbal invitations were also made by the neuroscience course directors at morning lectures and at the nearby required class activity. There were 12 different stations (Table 1) run by faculty, residents, fellows, technicians, and industry representatives. Ten stations were dedicated to the field of neurology.

**Table 1:** Stations at the Neurosciences Procedure Workshop

Procedure by specialty	Description	Led by
Neurology		
LP	Direction and practice performing LP on training models	Neurophysiology fellow
EMG/NCS	Students had brief NCS performed on them and the results explained	Neuromuscular fellow and technicians
Pupillometry	Practice using device and interpretation of results	Neurocritical care fellow
Dystonia muscle injection	Practice performing injections on arm anatomic muscle models	Movement disorders fellow and attending, Allergan representatives
Headache Botox injection	Practice performing injections on head models	Headache fellow and attending, Allergan representatives
Neuromodulation devices	Demonstration and use of neuromodulation devices used to treat headache	Headache fellow
DBS	Educational video of DBS implantation and live demonstration with a patient with Parkinson disease	Movement disorders attending
Adult neurologic examination	Demonstration and practice of correct neurologic examination techniques	Neurology clerkship director
Pediatric neurologic examination	Demonstration and practice of neurologic examination techniques unique to pediatric patients	Child neurology resident
Electroencephalography	Machine display and education	Epilepsy attending and technician
Neurosurgery		
EVD	Demonstration and practice using the neuronavigation system used to place EVDs	Neurosurgery attendings
Psychiatry		
ECT	Machine and educational video	Psychiatry attending

Abbreviations: DBS = deep brain stimulation; ECT = electroconvulsive therapy; EVD = external ventricular drain; LP = lumbar puncture; NCS = nerve conduction study.

These 10 stations were chosen to illuminate the variety of neurology subspecialties that are procedure heavy as well as common procedures and skill sets used by a general neurology physician (e.g., physical examination, lumbar puncture, EEG, EMG/nerve conduction studies, botulinum toxin for headache and dystonia, and pupillometry). Imaging was not included in the workshop due to the time and resources it would take to teach this skill set. The organizers reached out to faculty, trainees, and procedure managers from the following neurology subspecialties to participate in the workshop: epilepsy, movement disorders, neuromuscular, headache, pediatric neurology, general neurology, and neurocritical care. Because of time constraints in planning an extensive workshop and limitations in some subspecialties to showcase a procedure, the organizers did not send invitations to stroke, neuro-oncology, autonomic neurology, sleep medicine, and cognitive neurology. The number of booth participants, the size of the booth, and the scope of the procedures were determined by booth leaders.

Eight of these neurology-specific booths were hands-on booths, which included lumbar puncture with training models, pupillometry, EMG/nerve conduction study, headache and dystonia muscle injection with training models, neuromodulation headache devices, and the adult and pediatric neurologic examinations. There was also a live deep brain stimulation demonstration with a patient with Parkinson disease. The EEG booth compared the paper EEG machine with the one that is used today. One station was dedicated to neurosurgery, which demonstrated the neuronavigational system used for external ventricular drain placement. The psychiatry booth showcased electroconvulsive therapy equipment and videos of the procedure. Students were free to move between stations according to their time and interests.

### Workshop Objectives

The overall objectives of the workshop were the following: understand the diagnoses or neurologic complaints associated with each procedure, learn the basic techniques to perform the procedure or skill, and be able to perform the procedure after instruction.

### Assessment Method

Non-formal subjective assessment of the learners was fulfilled by the booth leaders through verbal interaction as is typically done on clerkships and teaching rotations. A formal assessment was completed with an exit survey to assess the learners' overall understanding of the procedure, the influence on their interest in the relevant specialties before and after attending the workshop, and satisfaction with each booth attended. In addition, there were questions gauging how each station helped the attendees better understand the procedure (eFigure 1, links.lww.com/NXG/A550). Interest in the clinical neurosciences was assessed using an interest scale (Likert), and interest preworkshop and postworkshop was dichotomized as responses of somewhat or very interested. Satisfaction with each booth was measured by a Likert scale, and helpfulness in understanding the procedure was defined as responses of slightly or very helpful. Students completed the survey after attending all or some of the booths. Qualitative data using written comments from attendees were also collected. An exit survey was chosen as the data collection method due to the simplicity and time required to encourage as many participants as possible to complete it. Surveys were administered by SIGN officers to ensure survey completion and prevent duplicate data.

### Standard Protocol Approvals

University institutional review board exemption was obtained because no identifying information was obtained with the surveys.

### Workshops Resources

The UT Southwestern Department of Neurology provided support for faculty time (H.D.) and resources to SIGN to organize the workshop. University faculty, trainees, and others donated their time and brought the required materials and instruments from their respective clinical sites where possible. The university simulation center loaned materials including neurologic examination instruments and lumbar puncture training models. Industry representatives from Allergan loaned the training models for headache and dystonia injections and ran the dystonia injection booth as volunteers. There was no financial incentive for their involvement, and no marketing content was delivered.

### Statistical Analysis

Statistical analysis of the survey results was conducted with SAS version 9.0. Change in student interest before and after the workshop was analyzed with a paired profile plot and Fisher exact test.

### Data Availability

Anonymized data that we did not share in this article can be made available on request from qualified investigators by contacting the corresponding author.

### Results and Assessment Data

Of the 482 preclerkship medical students, 111 attended the workshop, and 104 filled out the survey (94%). Although all medical students were invited to the event, most attendees (94%) were second-year students, and 6% were first-year students. Approximately 41% of the entire second-year class attended. Students expressed interest in a range of specialties (Table 2) and varying degrees of interest in pursuing neurology as a career.

**Table 2:** Current Specialty of Choice Among Attendees (n = 95^a^)

Specialty	No. of students
Adult neurology	6
Pediatric neurology	8
Neurosurgery	9
Psychiatry	5
Physical medicine and rehabilitation	2
Other neuroscience career	4
Other medical specialty	61
Total	95^a^

a Excludes 9 students who left the section blank or filled out the survey incorrectly.

**Figure 1:**
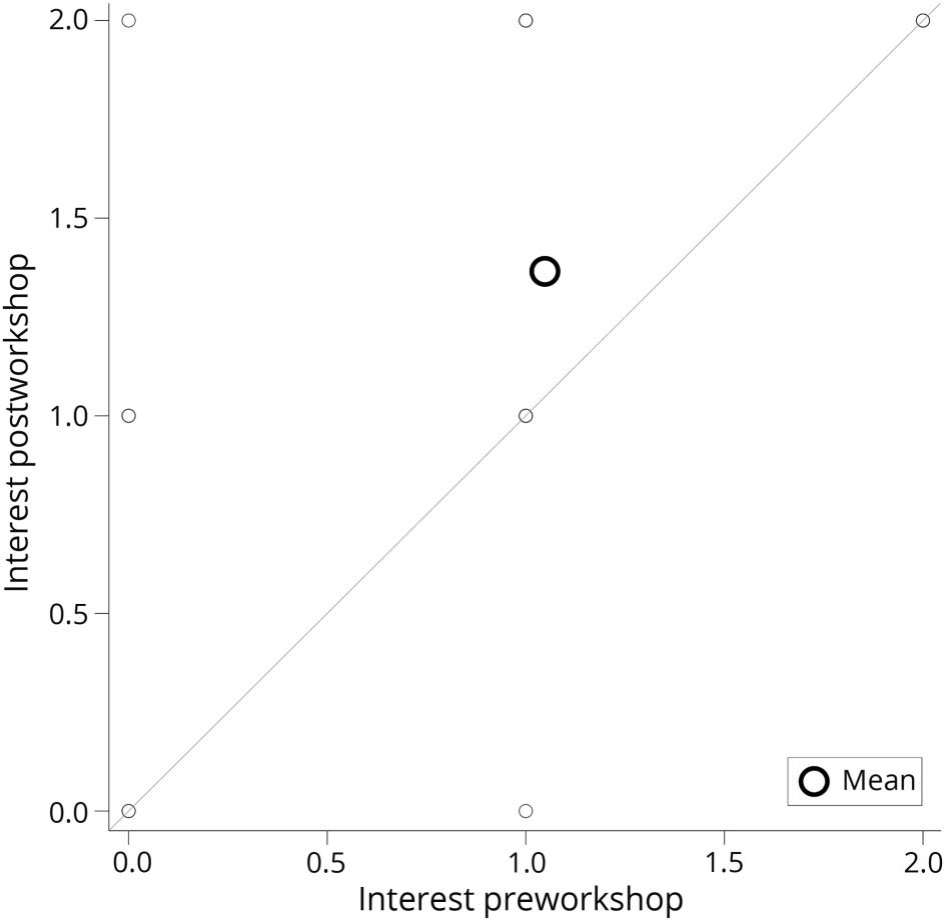
Change in Interest in the Clinical Neurosciences Pre- and Postworkshop Statistically significant change in interest in the clinical neurosciences pre- and postworkshop was seen with a *p* < 0.0001 (using Fisher exact test). Effect size was measured as d = 0.6346 indicating that the mean interest in the clinical neurosciences between the pre- and postworkshop groups was statistically significant. This is seen in the figure above where the mean interest (indicated by the large open circle) in the postworkshop group (y-axis) was significantly higher than the preworkshop group (x-axis).

Overall, there was an increase in self-reported interest in the clinical neurosciences, assessed using the Fisher exact test (*p* < 0.0001, Table 3) with an effect size of *d* = 0.6346 (Figure 1). Postworkshop, 99 of the students (95%) indicated that they were somewhat interested or very interested. Of this subset, 33 students (32%) noted an increased interest from the workshop, including 18 of the 22 students (82%) who had no prior interest. No change in interest in the neurosciences was reported by 70 students (67%). Only 1 student reported a decreased interest from the workshop.

**Table 3:** Interest in a Clinical Neurosciences Career Preworkshop and Postworkshop (n = 104)

		Postworkshop interest (no. of students)			
		None	Some	High	Total
Preworkshop interest (no. of students)	None	4	17	1	22
	Some	1	39	15	55
	High	0	0	27	27
	Total	5 (5%)	56 (54%)	43 (41%)	104 (100%)

Fisher exact test	
Table probability (P)	<0.0001
Pr ≤ P	<0.0001

All 12 booths were rated slightly or very helpful by most attendees who provided feedback for each booth (Table 4). For any given booth, there were several students who indicated that they did not attend the booth or who did not provide any response. Many students expressed positive comments about the workshop (Table 5), including a few students who expressed that they enjoyed the event, although they do not have interest in pursuing neurology as a career.

**Table 4:** Percentage of Attendees at Each Booth Who Rated It as Slightly or Very Helpful

Adult neuro examination	100% (25/25)
EMG	100% (43/43)
Botox and neuromodulation devices	98% (51/52)
Pediatric neuro examination	96% (26/27)
Deep brain stimulation	95% (35/37)
Dystonia muscle injection	94% (32/34)
Lumbar puncture	94% (45/49)
Electroconvulsive therapy	91% (31/34)
Pupillometry	90% (27/30)
External ventricular drain	88% (32/36)
Electroencephalography	80% (33/41)

**Table 5:** Student Feedback in the Comments Section (n = 69/104)

Type of feedback	No. of students
No written feedback	35
Expressed learning or experiencing something new at the workshop	22
Expressed that the event or a specific activity was interesting or fun	21
Expressed other positive comments about the event	17
Expressed neutral comments	9
Expressed positive comments about the clinical neurosciences	7
Expressed thanks	6

### Discussion and Lessons Learned

The neurosciences procedure workshop overall proved successful and popular among students who attended. A majority of students (95%) who attended either maintained or increased an interest in pursuing a career in the clinical neurosciences. For those students who did not have interest in the clinical neurosciences after attending the workshop, the event may have helped inform their choice, and even these students left positive comments about the experience.

Although data about the workshop's influence on eventual specialty choice are beyond the scope of this study, various studies have shown that a high-quality preclinical education in the neurosciences is a highly influential factor.^4,8-12^ This may be particularly true at schools where other exposures to a given specialty are limited. It may be presumed that the high attendance and positive feedback from students regarding our procedure workshop demonstrated a contribution to their overall preclinical neuroscience experience.

Mentors and quality personal interactions with providers in a given specialty have been discussed as another influence on specialty choice.^9,16^ The open structure of the workshop provided students facetime with specialty faculty members in a relaxed setting, and multiple students commented on this fact.

Self-perceived inadequacy with the content of neuroscience is a commonly recorded trend among students and providers coined as neurophobia. Some of this may be attributed to the complexity of the content of neuroscience in preclerkship education. In practice, however, neurology is often a hands-on specialty. In other specialties such as surgery, demonstration and training of procedures increases student and provider confidence in their abilities and stimulates interest in the specialty,^13,14^ and our study provides evidence that this is also true in the neurosciences. The majority of students in our study gained new understanding about each procedure, and students reported excitement about the tools and techniques used in neurology of which they were previously unaware.

Limitations to this study include the potential for recall bias due to the data being obtained as an exit survey and selection bias where those who chose to attend the workshop may have had overall higher interest in or openness to the neurosciences than those who chose not to attend. This is inherent to the event being an optional student activity, and making the event a required course activity may have yielded different results. There was also a selection bias toward the second-year medical student class, as they were in the preclerkship neuroscience course at the time and were the ones to whom the event was most advertised. Student interest in a specialty may be artificially heightened while students are taking the respective course block and does not necessarily translate to being more likely to choose that specialty for residency. Related to this, another limitation is the lack of data correlating the workshop with actual residency specialty choice. This would be difficult to assess due to the multitude of other factors involved in students' specialty choices including personal background, clinical or clerkship experiences, specialty lifestyle, mentors, perceptions of prestige or competitiveness, and so on.^4,8-11,17,18^ However, a survey of students who attended and eventually matched into the neurosciences could provide some data on this. In addition, our study lacks a control comparator such as a standard clinical or didactic experience in the neurosciences. This could be a future direction of research to better compare the utility of a hands-on experience in the neurosciences vs other teaching modalities. There may also be limited generalizability of the results to medical school sites with fewer neuroscience resources. Robust departments in the neuroscience specialties are not found at all medical schools (e.g., surgical epilepsy centers^19^), which would make it a challenge to put on such an event. For example, some medical schools do not have a required neurology clerkship, perhaps due to smaller neuroscience departments and clinical programs. However, not all the booths in our workshop are necessary to put on a similar event at another site. In addition, our study cannot draw conclusions about the reach to students of underrepresented minorities because such demographic data were not collected, but this would be a valuable future direction of study.

Although the procedure workshop was effective at increasing student interest in the clinical neurosciences, it is only one of many ways that schools can do so. The SIGN at our site also put on various other events during students' preclerkship education to increase exposure and opportunities in neurology. These included presentations of available research opportunities and contacts in the department, subspecialty job talks, case presentations intersecting neurology with the other organ-system preclinical courses (e.g., presentation of a CNS lymphoma case during the hematology course), and meet-and-greets with students, residents, and fellows. Other medical schools and student interest groups may consider planning similar activities to spread excitement for neuroscience.

A single hands-on procedure workshop improved medical student interest in the clinical neurosciences. Although its direct effect on specialty choice is beyond the scope of this study, preclerkship experiences such as a procedure workshop could be a useful addition to medical school curricula to foster interest in neurology and the clinical neurosciences.

## Appendix Authors

**Table:**

Name	Location	Contribution
Collin Sanderson	Department of Neurology, University of Texas Southwestern Medical Center, Dallas	Drafting/revision of the manuscript for content, including medical writing for content; major role in the acquisition of data; study concept or design; and analysis or interpretation of data
Khadijah Mazhar	Department of Neurology, University of Texas Southwestern Medical Center, Dallas	Analysis or interpretation of data
Mark P. Goldberg, MD, PhD	Department of Neurology, University of Texas Health Science Center, San Antonio	Drafting/revision of the manuscript for content, including medical writing for content, and analysis or interpretation of data
Shilpa Chitnis, MD	Department of Neurology, University of Texas Southwestern Medical Center, Dallas	Drafting/revision of the manuscript for content, including medical writing for content, and analysis or interpretation of data
Ryan Hays, MD	Department of Neurology, University of Texas Southwestern Medical Center, Dallas	Drafting/revision of the manuscript for content, including medical writing for content
Hina Dave, MD	Department of Neurology, University of Texas Southwestern Medical Center, Dallas	Drafting/revision of the manuscript for content, including medical writing for content; major role in the acquisition of data; study concept or design; and analysis or interpretation of data

## Glossary

SIGN: Student Interest Group in Neurology
